# Transnational Corporations as ‘Keystone Actors’ in Marine Ecosystems

**DOI:** 10.1371/journal.pone.0127533

**Published:** 2015-05-27

**Authors:** Henrik Österblom, Jean-Baptiste Jouffray, Carl Folke, Beatrice Crona, Max Troell, Andrew Merrie, Johan Rockström

**Affiliations:** 1 Stockholm Resilience Centre, Stockholm University, 106 91, Stockholm, Sweden; 2 Global Economic Dynamics and the Biosphere Academy Programme, Royal Swedish Academy of Sciences, PO Box 50005, 104 05, Stockholm, Sweden; 3 Beijer Institute of Ecological Economics, Royal Swedish Academy of Sciences, PO Box 50005, 104 05, Stockholm, Sweden; Aristotle University of Thessaloniki, GREECE

## Abstract

Keystone species have a disproportionate influence on the structure and function of ecosystems. Here we analyze whether a keystone-like pattern can be observed in the relationship between transnational corporations and marine ecosystems globally. We show how thirteen corporations control 11-16% of the global marine catch (9-13 million tons) and 19-40% of the largest and most valuable stocks, including species that play important roles in their respective ecosystem. They dominate all segments of seafood production, operate through an extensive global network of subsidiaries and are profoundly involved in fisheries and aquaculture decision-making. Based on our findings, we define these companies as *keystone actors * of the Anthropocene. The phenomenon of keystone actors represents an increasingly important feature of the human-dominated world. Sustainable leadership by keystone actors could result in cascading effects throughout the entire seafood industry and enable a critical transition towards improved management of marine living resources and ecosystems.

## Introduction

Globalization is increasing the economic power of transnational corporations in relation to national governments, but the effects of this gradual shift are largely unknown [1]. The world’s largest transnational corporations are highly connected and a small number of companies control a major part of global financial flows. This core of highly connected companies has been described as a “super-entity” of the global economy [2]. Conceptually, these companies may be analogous to keystone species in ecological communities. Keystone species have a profound and disproportionate effect on communities and ecosystems and determine their structure and function to a much larger degree than what would be expected from their abundance [3, 4]. The fact that individual actors can have a disproportionate impact on the environment is well known [5–7] and has recently been investigated in relation to greenhouse gas emissions [8]. While such “asymmetries” have been related to the keystone concept in general [9], this paper focuses on investigating the seafood industry in particular.

Globally increasing demand for seafood, a critical source of protein for human wellbeing, is shaping marine ecosystems and the harvest of wild capture fisheries and aquaculture production worldwide [10, 11]. Globalization has led to industry consolidation, with large and vertically integrated transnational corporations operating across entire supply chains from production through to retail [1, 12, 13]. These transnational seafood corporations play an important role in linking distant species and ecosystems to major markets and consumers [10]. At the same time their activities may influence important species and the dynamics and resilience of the ecosystems on which their seafood harvesting and production ultimately depend [14]. There is evidence that such human exploitation of natural resources has contributed to cascading ecological effects and regime shifts, with resulting system-wide changes [15–18].

The interplay of transnational corporations and ecosystems across geographical scales may represent an important feature of the global social-ecological system [19, 20]. Although there is a growing recognition that human activities dominate a globalized and interconnected planet (often referred to as the Anthropocene [21, 22]), the role of global actors like transnational corporations has received limited attention in studies of ecosystem management and in particular marine ecosystem management. Existing analyses of global fisheries operations have focused on the role of individual major countries, rather than transnational corporations, including the former Soviet Union [23] and China [24], two of the largest fishing nations in the world.

In this paper, we analyze whether or not a keystone pattern [9] can be observed in the relationship between transnational corporations and marine ecosystems globally, from a combined ecological, economic and policy perspective. If such actors operate analogous to keystone species, they would not only have a disproportionate ability to steer the direction of the seafood industry but also to shape the world’s marine ecosystems and how they are managed.

## Materials and Methods

In order to investigate the pattern of revenues for seafood companies, we examined two recent global fishing industry reports [12, 13] and calculated the average annual revenues of the 160 largest companies for 2012. Changes in consolidation over time, were assessed by comparing revenues from 2012 with those from 2007 (corrected for inflation), using data assembled by the Organisation for Economic Co-operation and Development (OECD) [10]. We sampled the top ten per cent of the 160 largest companies and excluded the ones engaged exclusively in distribution, trading, import, export, processing or retail. The remaining companies were involved in all segments of seafood production (both wild capture fisheries and aquaculture), including whitefish, tuna, small pelagic species, salmon, shellfish, fishmeal, fish oil and aqua feeds (salmon, shrimp and whitefish feeds combined). We used FIS (Fish Information & Services) [25] to determine the total number of registered fishing and aquaculture companies.

In the following, we describe; how we documented the global activities of these companies, including their catch of wild species and production of aquaculture species and the economic value of these species; how these catch and production volumes were linked to global volumes; and the extent to which these companies participate in fisheries and aquaculture policy and management processes.

### The global activities of the selected companies

To document the activities of the companies we studied their web pages, annual/quarterly reports and company presentations (S1 Table). We also engaged in direct correspondence with company representatives at headquarters locations (in Japan, Korea, Hong Kong, Thailand, Norway, Spain, and the USA) and several of their individual subsidiaries (in Australia, Japan, New Zealand, Norway, Sweden, Thailand and the USA). Several recent global fishing industry reports (S2 Table) and information derived from subscriptions to *IntraFish (Fishing News*), *FIS*, *Undercurrentnews*, and *Minato*-*Tsukiji* (S2 Table) complemented the information obtained directly from the companies. We also conducted targeted (i.e. company specific) searches using multiple other media sources: *atuna*, *the Fish Site*, *Seafood Source*, *Food Business Review*, *Shrimp News International*, *The Center for Public Integrity* and *Bloomberg* (S2 Table).

We corresponded with international organizations, including the Food and Agriculture Organization of the United Nations (FAO, Italy), OECD (France), the Commission for the Conservation of Antarctic Marine Living Resources (CCAMLR) Secretariat (Australia) and the Marine Stewardship Council (MSC, United Kingdom, Singapore), as well as with fisheries management agencies (in Alaska, Chile, Namibia, Peru, the United Kingdom), journalists (Argentina, United Kingdom), environmental NGOs (Hong Kong, Korea, New Zealand, Norway, Sweden) and scientists (Canada, Chile, France, Japan, Peru, Uruguay, the USA) for further company specific information. We also used additional data from *Fishsource* [26] as well as a number of region- (Alaska, Japan, Korea the Southern Ocean, the North East Atlantic), species- (tuna, shrimp, toothfish) and segment- (whitefish) specific information sources (S2 Table).

We systematically documented the number of species handled by each company and the number of countries and territories to which they are connected (through direct operations, subsidiaries, sales or procurement offices), using the information sources described above and in S1 and S2 Tables.

### Wild species considered and their ecological role

To estimate the role of these largest companies in global fisheries catches, we investigated their activities in relation to the largest and economically most important wild-capture stocks, representing whitefish, tuna, and small pelagic species [27]. We collected information on the catch activities of the companies with respect to the largest wild fish stocks in the world, including the stock with the largest registered catch volume in 2012: Peruvian anchovy *Engraulis ringens* and the stocks with the 2^nd^ largest (Alaska pollock *Theragra chalcogramma*), and the 3^rd^ and 6^th^ largest (skipjack tuna *Katsuwonus pelamis* and yellowfin tuna *Thunnus albacares*) catch volumes. Atlantic herring *Clupea harengus* was the species with the 4^th^ largest catch volume in 2012, but as this species is widely dispersed over the entire North Atlantic, and was often reported together with other small pelagic species by the investigated companies, we instead pooled information on small pelagic species (Atlantic mackerel *Scomber scombrus*, Atlantic horse mackerel *Trachurus trachurus*, herring, sprat *Sprattus sprattus*, capelin *Mallotus villosus*, blue whiting *Micromesistius poutassou*, sandeel *Ammodytes* spp. and Norway pout *Trisopterus esmarkii*) from the entire North East Atlantic region (FAO Area 27, encompassing e.g., the North, Norwegian, Barents and Baltic Seas). Due to a lack of data availability and ambiguity in the use of the name “mackerel”, we were unable to include Chub mackerel *Scomber japonicas* (the stock with the fifth largest catch volume in 2012).

These selected stocks include species that, by volume and value, dominate their respective segments of seafood production (Alaska pollock dominates the white fish market [28], whereas skipjack and yellowfin tuna dominate the tuna market [28], and Peruvian anchovy dominates the market for small pelagic species used for production of fishmeal and fish oil [28]). Owing to data availability, we were also able to include two additional species of global importance: Namibian hake *Merluccius* spp, and toothfish (Patagonian toothfish *Dissostichus eleginoides* and Antarctic toothfish *D*. *mawsoni* combined). Namibian hake is (together with Argentinean hake) the second largest (by volume) white fish species [28] while toothfish represents the most valuable species in the Southern Ocean [29].

The key ecological role of the species included in this study has been extensively described elsewhere. For information on Peruvian anchovy, see e.g., [30], Alaska pollock [31], skipjack and yellowfin tuna [32, 33], herring and the other small pelagic species [34–36], toothfish [37, 38] and Namibian hake [39, 40].

### Aquaculture species considered

We also investigated the production of predatory species in aquaculture, including a) global salmon production (the most valuable segment of seafood production [28]), b) shrimp (the most traded seafood commodity in value terms, with an average of 30% growth in volumes, per year, during the last ten years [28])—we specifically included Vannamei shrimp (*Penaeus vannamei*), or whiteleg shrimp, the largest component of shrimp aquaculture (accounting for 71% of global marine shrimp aquaculture production volumes in 2010 [28]) and c) farmed Bluefin tuna *Thunnus* sp. (one of the most valuable and controversial fish species [41]). Aquaculture of predatory fish species and high value shellfish species (such as salmon, tuna and shrimp) are particularly dependent on marine ecosystems due to the high level of inclusion of fishmeal and fish oil in their feeds. Finally, we quantified the companies’ production of fishmeal, fish oil and aqua feeds.

### The economic value of species caught or produced

The economic value of the investigated wild fish stocks and aquaculture species in 2012 was determined using species specific values (USD/ton), derived from a seafood investment report [28], expert estimates (global experts who work with the corresponding stocks on a daily basis) and FAO data [42]. For small pelagic species in the North East Atlantic and for Namibian hake, we used general values [42] of herring and hake, respectively.

### Linking company specific data to global volumes

We used data on quotas and catches produced in 2012 (or 2013 in one case) by the respective company and its present subsidiaries, accounting for known overlap between companies, to avoid double counting the proportion of each stock caught or produced. We exclusively relied on published and official data, combined with information provided directly by the respective companies, without accounting for estimates of illegal or underreported catches or production volumes, for any of the species included. Some companies were also involved in the production of feeds for terrestrial animals, but these volumes were not included in our analysis. Company specific volumes were compared to global and regional volumes, using data from the FAO [42–45], or other relevant regional authority (S2 Table), with support from the Statistics and Information Branch at the FAO Fisheries and Aquaculture Department.

### Participation in fisheries and aquaculture policy and management

We studied participation in globally relevant institutions as a proxy for the potential of the companies to influence fisheries and aquaculture policy and management. We quantified the number of occasions that a company was registered as a participant during the meetings of the thirteen Regional Fisheries Management Organizations (RFMOs). These organisations together represent an established mechanism for managing fisheries, including stocks of tuna, pollock, toothfish and some small pelagic species. We used the most recent meeting documents from the main governing body of these RFMOs, including five tuna RFMOs: the Commission for the Conservation of Southern Bluefin Tuna (CCSBT), Inter-American Tropical Tuna Commission (IATTC), International Commission for the Conservation of Atlantic Tunas (ICCAT), Indian Ocean Tuna Commission (IOTC) and the Western and Central Pacific Fisheries Commission (WCPFC), and eight non-tuna RFMOs: Commission for the Conservation of Antarctic Marine Living Resources (CCAMLR), Convention on the Conservation and Management of Pollock Resources in the Central Bering Sea (CCBSP), General Fisheries Commission for the Mediterranean (GFCM), North East Atlantic Fisheries Commission (NEAFC), North Atlantic Salmon Conservation Organization (NASCO), Northwest Atlantic Fisheries Organization (NAFO), South-East Atlantic Fisheries Organization (SEAFO) and South Pacific Regional Fisheries Management Organization (SPRFMO) (S2 Table). We also reviewed company-specific membership in global industry organisations present in several of these RFMOs.

In addition we evaluated the role of the investigated companies in establishing, or the extent they were members in three international organizations identified as important industry initiatives for aquaculture management and certification [46]—namely, the Global Aquaculture Alliance (GAA), the Aquaculture Stewardship Council (ASC) and the Global Salmon Initiative (GSI). Finally, we assessed company membership in the Marine Ingredients Organization (IFFO), the central organization that represents companies producing fishmeal, fish oil and feeds.

## Results

The investigated companies were all identified as playing a disproportionate role in marine social-ecological systems. They a) dominate global seafood volumes and revenues, b) are globally connected through subsidiaries and other networks of operations, c) dominate globally relevant segments of seafood production, and d) are represented in global fisheries and aquaculture policy and management. When combined, these aspects provide them with a disproportionate role in the global seafood production industry and a disproportionate ability to influence the dynamics of marine ecosystems worldwide. We label these corporations *keystone actor*s.

### Disproportionate revenues and globalization

**Fig 1:**
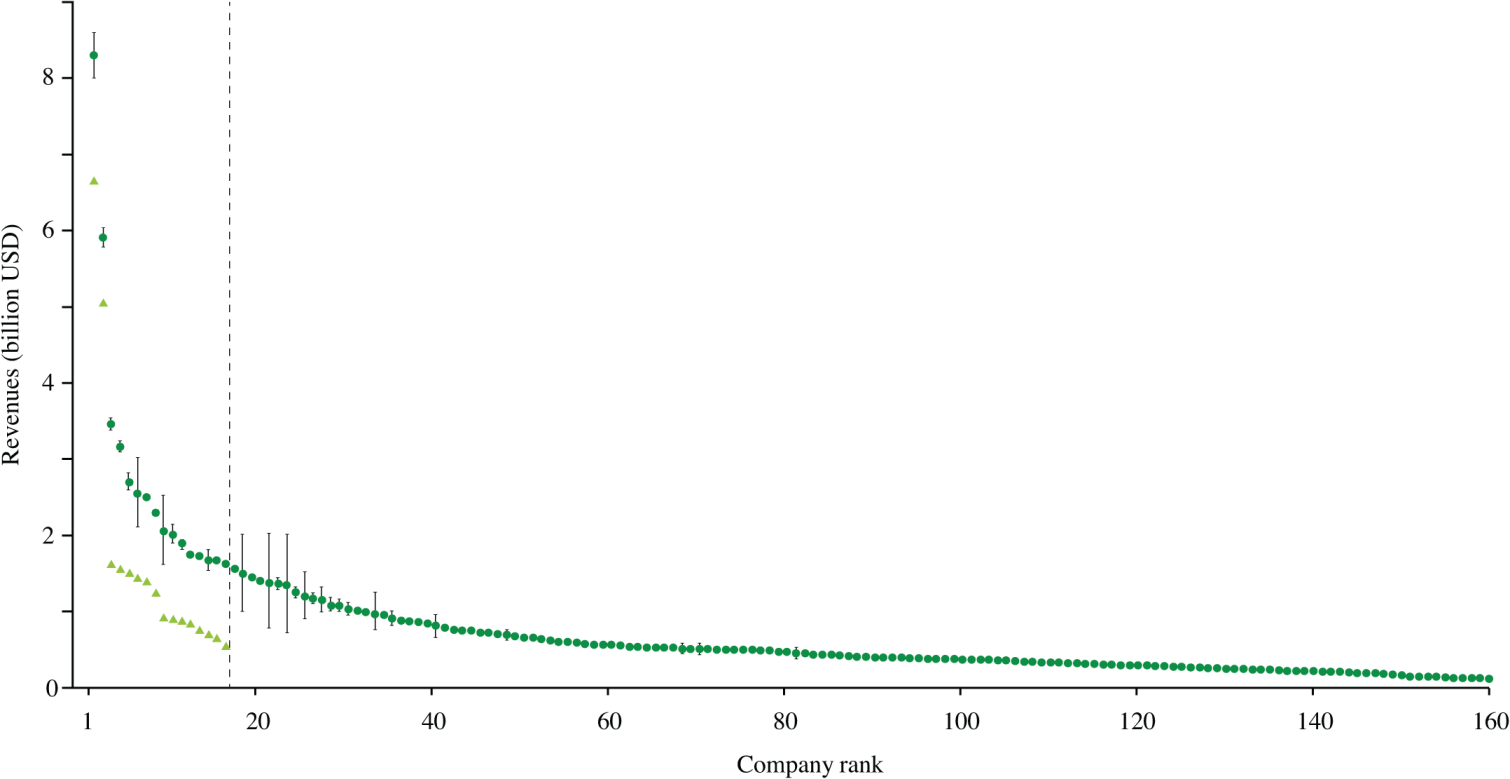
Revenues of the 160 largest seafood companies. Revenues of the 160 largest seafood companies in 2012 [12, 13] (circles, data show as mean ± s.e.m.) with the top ten per cent indicated by the dashed line, and corresponding revenues of the top 16 seafood companies in 2007 [10] (triangles). Maruha Group (ranked 1^st^ in 2007) and Nichiro Corporation (ranked 5^th^ in 2007) merged in 2008 to form Maruha Nichiro (ranked 1^st^ in 2012). Pacific Andes (ranked 15^th^ in 2007) acquired China Fishery Group Limited (ranked 23^rd^ in 2007) and is currently the 9^th^ largest seafood company in the world.

The average annual revenues of the 160 largest companies in 2012 (Fig 1) exhibit a distinct keystone pattern (cf. [9]), where the top ten per cent account for 38% of total revenues. Their annual revenues also increased substantially between 2007–2012 (Fig 1) illustrating the on-going consolidation process within the global network of seafood production.

Thirteen of the sixteen largest companies operate directly in marine ecosystems through their harvesting and farming activities around the world, with headquarters in Norway (four companies), Japan (three), Thailand (two), Hong Kong, Korea, Spain and the USA (one each), see (Table 1). The combined annual revenues of these thirteen companies (representing 0.5% of 2250 registered fishing and aquaculture companies worldwide) correspond to 18% of the global value of seafood production in 2012 (US$ 252 billion).

**Table 1:** The investigated thirteen seafood companies.

Company	Headquarters	Market
Maruha Nichiro	Tokyo, Japan	A globally operating seafood company active in most segments of seafood production
Nippon Suisan Kaisha (Nissui)	Tokyo, Japan	A globally operating seafood company active in most segments of seafood production
Thai Union Frozen Products	Samutsakorn, Thailand	The world’s largest canned tuna producer and fifth largest shrimp farmer (2011)
Marine Harvest	Bergen, Norway	The world’s largest salmon producer and the most actively traded stock in the seafood sector
Dongwon Group	Seoul, South Korea	A national (75% of Korean canned tuna market share) and world leading tuna producer (together with Thai Union)
Skretting	Stavanger, Norway	A leading salmon feeds producer
Pescanova	Pontevedra, Spain	The world’s second largest shrimp producer and the largest fishing company in the European Community
Austevoll Seafood	Storebø, Norway	The world’s largest fishmeal company and second largest salmon producer
Pacific Andes	Hong Kong, China	The world’s second largest fishmeal producer
EWOS	Oslo, Norway	A leading salmon feeds producer
Kyokuyo	Tokyo, Japan	Similar to Maruha Nichiro and Nissui, but with relatively more limited operations
Charoen Pokphand Foods (CP Foods)	Bangkok, Thailand	The world’s largest shrimp farmer and the largest shrimp feeds producer
Trident Seafood	Seattle, USA	The largest seafood company in North America

**Fig 2:**
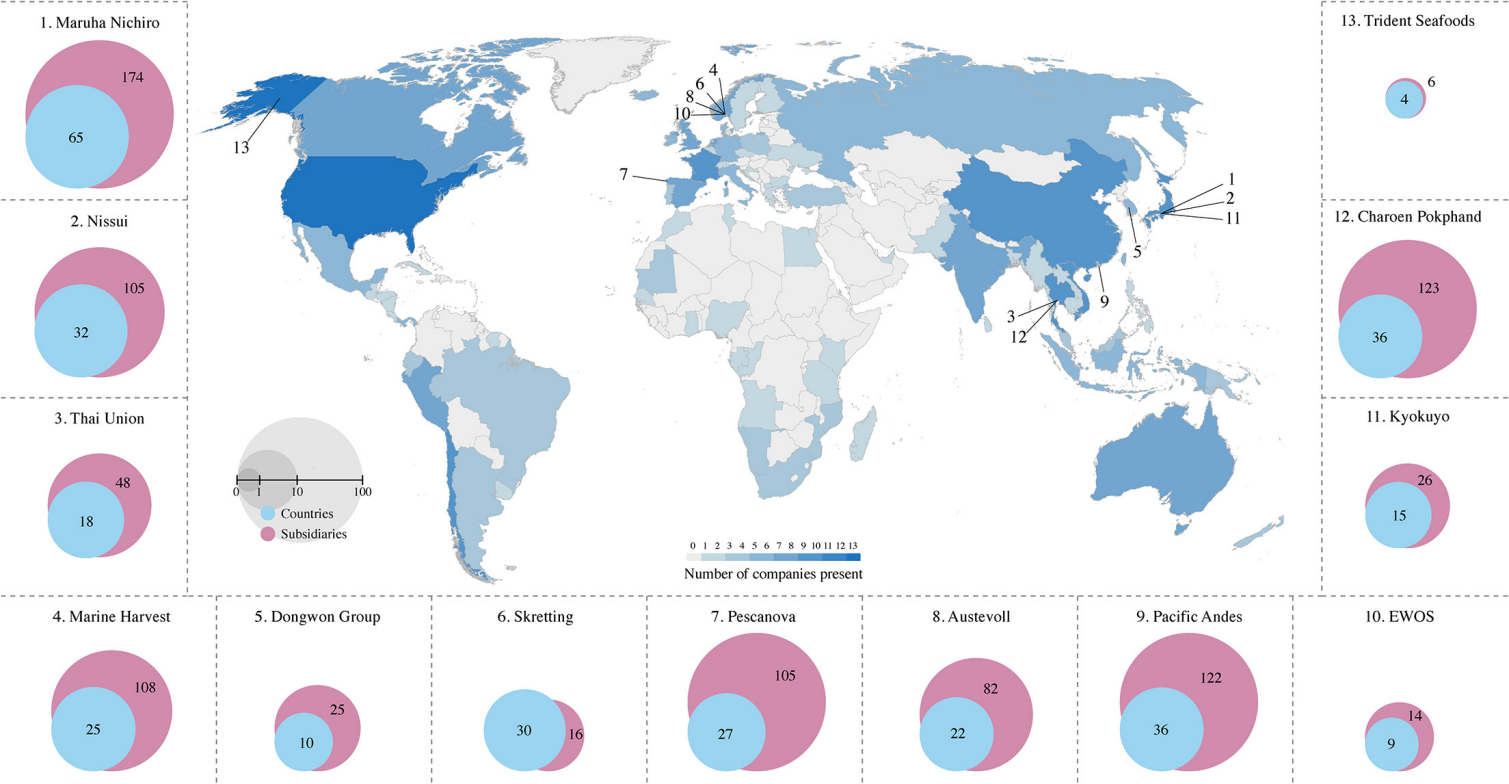
Global networks of operations. Heat map illustrating the number of keystone actors operating in each country and the respective number of countries in which each company operates (blue circles) as well as the total number of subsidiaries of that company (purple circles). Company headquarters locations are indicated by the corresponding numbers on the map.

These transnational corporations are catching, farming and handling more than 208 species from 974 subsidiaries and associates operating in 102 countries and territories (Fig 2). They are each highly connected and act as key nodes in the global seafood production system.

### Global volumes caught and produced

We conservatively estimate that these companies combined handle between 9 and 13 million tons of wild-caught fish annually (shellfish included), corresponding to 11–16% of the total global marine catch (S3 Table). The catches of the three Japanese companies alone (including their international subsidiaries) are of the same order of magnitude as the total volume for the entire country of Japan, one of the world’s largest seafood producing countries [45].

**Fig 3:**
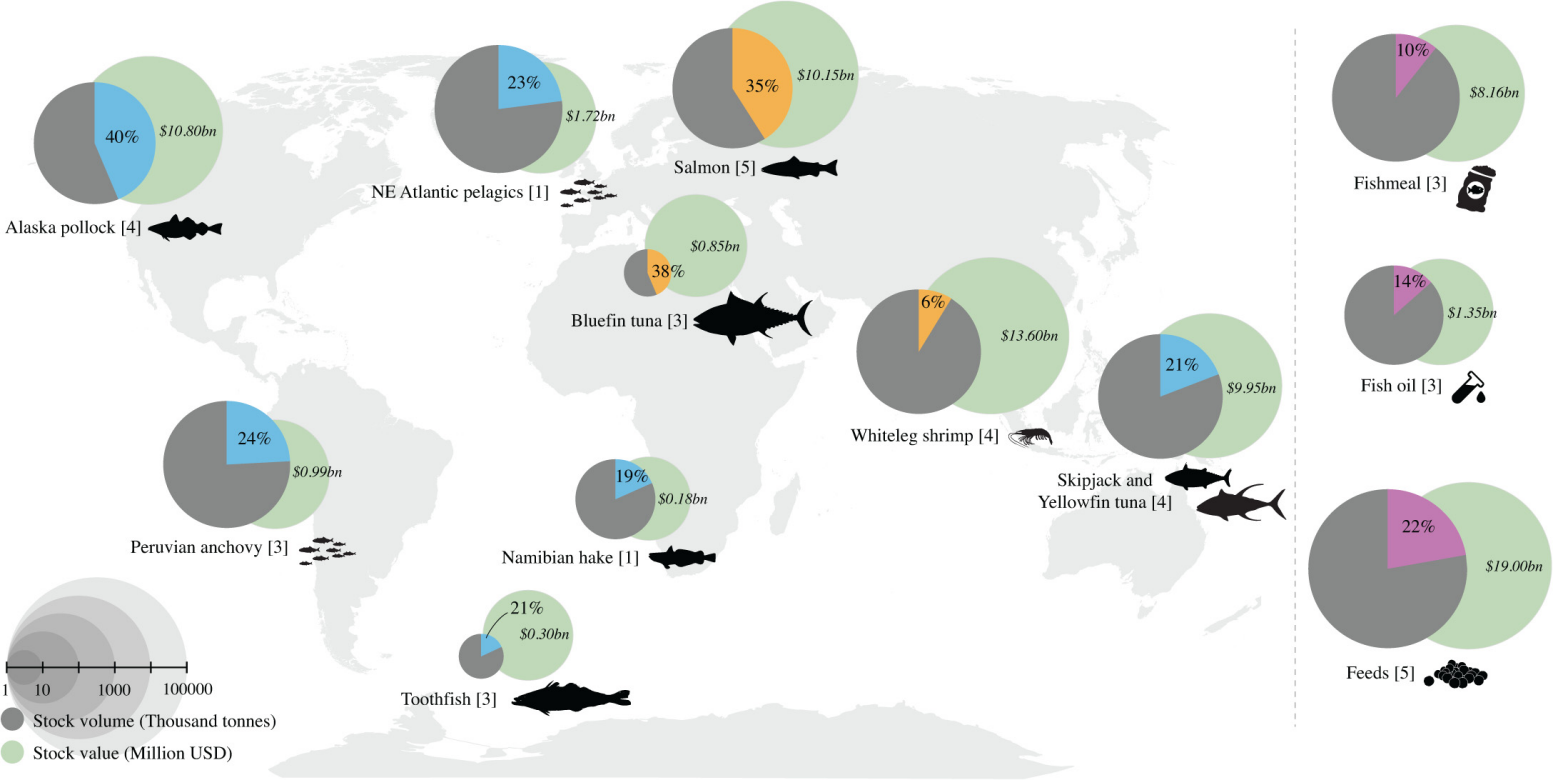
Regional fisheries of global relevance. Globally important wild fish stocks by volumes (grey circles with blue wedges), aquaculture production volumes (orange wedges), and global fishmeal, fish oil and aqua feeds (salmon, shrimp and whitefish feeds combined) volumes (purple wedges), and their corresponding economic value (green circles). The proportion of each stock controlled by the keystone actors is indicated by the size of the wedge. The number of companies active in each stock is shown within brackets.

The keystone actors together control 19–40% of several of the world’s largest or most valuable capture fisheries (Fig 3), including three of the most important wild-caught stocks used for human consumption: Alaska pollock (the largest whitefish stock), skipjack and yellowfin tuna (the largest tuna stocks used for the canned tuna and sashimi markets). Examples of regionally important food fish species include toothfish and Namibian hake (Fig 3). These companies also catch large volumes of small pelagic species, including Peruvian anchovy (the largest wild capture fishery in the world) and Atlantic herring, both important ingredients in fishmeal, fish oil, and aqua feeds. They produce 10% and 14% of global fishmeal and fish oil volumes respectively and 22% of global aqua feeds (including 68% of the salmon feeds and 35% of the shrimp feeds, S3 Table).

Several of the companies produce high value predatory species in aquaculture, including 35% of global salmon and trout volumes and 38% of farmed bluefin tuna (Fig 3). The studied companies also include the three largest producers of shrimp in the world. Salmon, bluefin tuna and shrimp represent the best performing segment of the seafood industry, one of the most valuable species and the most traded seafood commodity (measured in value), respectively. Combined, these thirteen companies dominate the catch and production of the major species globally, both in terms of quantity and value.

### Participation in fisheries and aquaculture policy and management

We investigated to what extent the globally connected actors participated in policy processes and found that three of the investigated companies were among the few (10%, n = 145) that were identified as active in more than one regional fisheries management organization (RFMO). They were active either as observers or as members of national delegations. The Korean company was the most active company overall, participating in six RFMOs. In addition to direct representation by the parent company and its subsidiaries, keystone actors are also indirectly participating in RFMOs through influential industry organizations (Table 2). Keystone actors are also working directly with governments in a number of countries, including in Namibia, to ensure continued access to Namibian hake [41], or with small-island developing states in the Western Central Pacific, to secure access to tuna resource, in a region where almost 60% of the global tuna catch is taken [47].

**Table 2:** Global industry organizations actively engaged in fisheries and aquaculture policy and management.

Name of organization	Focus of organization	Role of keystone actors
*Wild capture fisheries*		
The International Seafood Sustainability Foundation (ISSF) [48]	Conservation and sustainable use of tuna resources. ISSF was established in 2009 and sets industry sustainability standards that aim to avoid by-catch and reduce Illegal, Unreported and Unregulated (IUU) fishing [46].	Founded by several tuna fishing companies, including Starkist (currently owned by Dongwon Group) as well as MW Brands and Chicken of the Sea (currently owned by Thai Union Frozen Products) [46].
Organization for the Promotion of Responsible Tuna (OPRT) [49]	Sustainable use of tuna resources. Established in 2000, OPRT represents fishing operators, traders, distributors and consumers, including e.g. The Korean Overseas Fisheries Association and the Japanese Tuna Fisheries Co-operative Association.	Korean tuna vessels are represented in OPRT through the Korean Overseas Fisheries Association. The Korean keystone actor (Dongwon Group) owns 15% of the vessels in OPRT flagged to Korea (n = 148). Maruha Nichiro is represented in OPRT through its subsidiary Taiyo A&F. Co. [49].
*Aquaculture and feeds*		
The Global Aquaculture Alliance (GAA) [50]	Sustainable aquaculture production. Established in 1997. Its Best Aquaculture Practices (BAP) certification, applicable to hatcheries, farms, processors and feed mills, is the most widely recognized sustainability label for farmed seafood [46].	The two Thai keystone actors (Thai Union Frozen Products and Charoen Pokphand Foods), as well as Pescanova (Pescanova USA) are among its governing members [50].
Aquaculture Stewardship Council (ASC) [51]	Global standards for responsible aquaculture. Founded in 2010, ASC is involved with aquaculture producers, seafood processors, distributors, conservation groups and consumers.	Nutreco (parent company of Skretting) and Marine Harvest both have representatives on the ASC Supervisory Board [51].
The Global Salmon Initiative (GSI) [52]	Sustainability of salmon production. GSI was established in 2013 and focuses particularly on feeds, to reduce disease and nutrient loadings, as well as other environmental and social impacts [46].	Two Norwegian keystone actors: Lerøy Seafood (a subsidiary of Austevoll) and Marine Harvest, were among the primary founders of the initiative [46].
The Marine Ingredients Organization (IFFO) [53]	Represents and promotes the fishmeal, fish oil and wider marine ingredients industry. Founded in 2001, IFFO is represented in a number of international policy making forums where it aims to promote these industries and work towards sustainable supplies in the future. IFFO has developed a Global Standard for the Responsible Supply of fishmeal and oil (IFFO RS) [53].	Nine keystone actors are direct or indirect members of IFFO, including Austevoll (through its subsidiaries Austral, Alimentos Marinos and Marfood), EWOS, Skretting, Marine Harvest, Maruha Nichiro, Trident, Nissui (through its subsidiary UniSea), Charoen Pokphand Foods and Pacific Andes [53, 54].

Eleven out of the thirteen companies were also actively engaged in the development of aquaculture management and certification processes, through their central role in one or several industry organizations that are actively working to influence the global context of aquaculture production (Table 2).

## Discussion

We have identified a small set of *keystone actors* that together dominate global seafood revenues. These companies play a critical role in increasing the connectivity in seafood networks and thereby operate as key nodes in what we here refer to as a global social-ecological system. This increasing connectivity is a recent and rapidly evolving phenomenon (Fig 1, S4 Table) and several of the identified actors are expected to lead future seafood industry consolidations [28]. Recent studies of complex system dynamics illustrate how increasing connectivity can facilitate critical transitions, including both positive change or unwanted collapse [15].

The identification of keystone actors can have substantial implications for fisheries and aquaculture policy and management. The investigated companies play a central role in relation to global fisheries catch volumes and dominate several of the world’s largest wild capture fisheries. The major wild caught species harvested by these companies are not only globally important resources for the seafood industry and consumers, but these species all individually play important roles in marine ecosystems (e.g. operating as predators or prey) and contribute to the structure, function and resilience of their respective ecosystems. Fishing for such species can have both direct and indirect effects on associated species and ecosystems. Skipjack and yellowfin tuna fishing using fish aggregation devices (FADs) or long-lines generates bycatch of juveniles and associated vulnerable species, including bigeye tuna *Thunnus obesus*, sharks, sea turtles and seabirds [55, 56]. Fishing can have a direct effect on small pelagic fish stocks [57] and large-scale ecological cascading effects on seabirds have been documented to result from the reduction in abundance of small pelagic species, e.g. in the North East Atlantic [35, 58]. Additional cascading ecological effects can be expected from the depletion of other species in our sample, as these species have all been identified as critically important for the structure and function of the ecosystems in which they are found [30–40].

The production of predatory fish in aquaculture is directly connected to marine ecosystems through the inclusion of wild fish in feeds (primarily small pelagic species). Salmon and shrimp are major consumers of aqua feeds (18% and 20% of global production volumes respectively [28]). However substantial efforts have been made to reduce the dependency of wild fish (particularly in salmon feeds [46]) by increasingly including plant protein sources and fisheries byproducts [59]. On the other hand bluefin tuna is still extensively dependent on wild fish in feeds (requiring >12 kg of wild fish per kilo of tuna produced [60]). Aquaculture operations may, in addition, have substantial effects on associated ecosystems, e.g., through nutrient pollution or acting as a vector for the spread of disease [61–63].

Not only do keystone actors have the ability to shape ecosystems—they also actively participate in policy-making. The ability of non-state actors, such as transnational corporations, to influence policies has been directly correlated to their degree of participation in global governance [64]. We have shown that keystone actors are active in countries worldwide and in global and regional fisheries and aquaculture policy and management activities. This means that they can potentially have a direct or indirect influence on future harvesting and production patterns as well as in designing policies (see e.g., [65, 66] for a description of the critical importance of the fishing industry in developing policies for reducing IUU fishing in CCAMLR).

Globally networked and vertically integrated companies, with an ability to influence policy-making, are resilient to disturbances that critically affect the survival of smaller companies, including financial system crises and instability, currency fluctuations, increasing fuel prices or changing fish stock dynamics [1]. The global connectivity of keystone actors (Fig 2) provides them with a unique overview that enables them to know how, when, where, and with which company to strategically prioritize harvesting and sourcing activities. As keystone actors are critically dependent on a continuous supply of marine products, such global scanning ability [1] ensures efficiency of production and consistency in resource supply. Keystone actors have historically increased their connectivity, analogous to the “rich-get richer” dynamics in other real world networks [67], through strategic mergers with major market or quota holders or via direct acquisitions (Fig 1 and S4 Table). Such increasing connectivity builds company resilience by adding value to the specific niche the company occupies, resulting in higher degrees of vertical integration and efficiency gains [1], while maintaining the flexibility to deal with changing situations and collapsing stocks (thereby increasing the prospects for substitution e.g. between different white fish stocks [68]).

As an example, *Pescanova*, the 7^th^ largest company in 2012, went bankrupt in 2013, but was, due to the resilience resulting from its global connectivity and diversification of activities (active in wild capture fisheries worldwide as well as in aquaculture), able to maintain its operations and trading activities despite the bankruptcy [69]. Other examples illustrating the ability of keystone actors to adapt to, or transform, in the face of crises, include Japanese keystone actors that adapted to widespread implementation of Exclusive Economic Zones (EEZs) in the 1970s by initiating joint ventures with, and acquisitions of, companies overseas (S4 Table) or, recent innovations in the aquaculture industry resulting from crises associated with infectious viral diseases and other sustainability challenges [28]).

The current global sustainability challenge in fisheries [70] is therefore not the first challenge confronting these keystone actors. Their institutional memory has provided them with experiences of how to deal with and direct the shift in fishable species, worldwide depletion of predatory fish stocks and the rise of aquaculture [11, 70].

Nation states have traditionally formed the basis for governance of fisheries resources and the majority of existing institutions are designed around this assumed reality, as are global fisheries statistics. Our study reframes the responsibility for fishing in terms of transnational corporations, illustrating that 13 companies handled around 10 million tons of wild capture fish in 2012, whereas only 23 countries caught >1 million tons of wild fish that year [45]. Several fishing companies are thus larger than most nations and at the same time take part in decision-making bodies for these resources. Previous studies have highlighted how an interconnected global society is moving faster and faster [10, 71]. Here, we have underlined the institutional challenges of the Anthropocene [72] and identified how keystone actors act as a central feature in this new global dynamic.

Fishing industry firms can play a key role for sustainability [29] but keystone actors have yet to assume this responsibility at the global scale. Pressure to engage in sustainability may come from governments, consumers, employees, competitors, investors or financial institutions [73]. The incentives for globally operating, profit driven and “biosphere dependent” companies to address sustainability concerns may vary. The annual (and in some cases sustainability-) reports of the investigated companies (S1 Table) illustrate that there is variation in the extent to which these companies regard sustainability as a business strategy. A number of companies have comprehensive sustainability strategies, whereas others appear less advanced in this respect. Active leadership in sustainability initiatives by the identified keystone actors could result in dramatic cascading effects throughout the entire seafood industry—potentially enabling critical transitions towards improved management of fish stocks and marine ecosystems globally. An analogous cascading effect occurred in the global retail industry after a few predominant actors decided to exclusively sell Marine Stewardship Council (MSC)-certified seafood products, which triggered other major retailers to follow their example [74].

## Conclusion

Globalization has resulted in the emergence of a small number of resilient transnational seafood corporations, operating across the world. Such *keystone actors* are critical in shaping the future direction of seafood production and as a consequence, the future of marine ecosystems. There is mounting evidence that similar keystone actors exist in other sectors [8, 75]. Based on our analysis, keystone actors are defined by the following characteristics: a) they dominate global production revenues and volumes within a particular sector, b) control globally relevant segments of production, c) connect ecosystems globally through subsidiaries and d) influence global governance processes and institutions. We propose that the phenomenon of keystone actors represents a critical feature of the Anthropocene, with high relevance for sustainable management of natural resources and the environment.

## Supporting Information

S1 Table Company specific references and web pages. (DOCX)

S2 Table Additional sources of information. (DOCX)

S3 Table Stock volumes handled by the studied companies, including the estimated total wild catch and the total catch for key segments in global seafood. (DOCX)

S4 Table History and vertical integration of the thirteen largest seafood producers. (DOCX)
