# Supplementary material for: Transnational Corporations as ‘Keystone Actors’ in Marine Ecosystems
Source: PLoS One. 2015 May 27;10(5):e0127533. doi: 10.1371/journal.pone.0127533 (PMC4446349; doi:10.1371/journal.pone.0127533)
Supplement: S1 Table — (DOCX) [file pone.0127533.s001.docx]

**Supporting Information Table S1.** Company specific references and web pages

| **Company** | **References** | **Webpages** |
| --- | --- | --- |
| Maruha Nichiro | [1-3] | [4-13] |
| Nippon Suisan Kaisha | [14-23] | [24-29] |
| Thai Union Frozen Products | [30-35] | [36] |
| Marine Harvest | [37-44] | [45] |
| Skretting | [46-49] | [50,51] |
| Pescanova | [52-59] | [60,61] |
| Dongwon | [62-64] | [65,66] |
| Austevoll | [67-73] | [74-81] |
| Pacific Andes | [82-90] | [91-93] |
| Trident Seafoods | [94,95] | [96] |
| Kyokuyo | [97-99] | [100-105] |
| EWOS | [106-114] | [115] |
| Charoen Pokphand Foods | [40,41,116-120] | [121] |

**References**

1. Anonymous (2012) Corporate Profile 2012. Tokyo: Maruha Nichiro.

2. Anonymous (2013) Financial Statement For the year ended March 31, 2013. Tokyo: Maruha Nichiro Holdings, Inc.

3. Ettefagh S (2013) 2012 Final Report Unalaska Fleet Cooperative. Wrangell: Sylver Fishing Company.

4. Anonymous (2014) Maruha Nichiro Corporation. Tokyo: Maruha Nichiro Corporation.

5. Anonymous (2014) Taiyo A&F Co., Ltd. Taiyo A&F Co., Ltd.

6. Anonymous (2014) transOCEAN products. Bellingham: Trans-Ocean Products.

7. PeterPan (2014) PeterPan Seafoods, Inc. Seattle: Peter Pan Seafoods.

8. Westward (2014) Westward Seaffoods. Seattle: Westward Seaffoods Inc.

9. Anonymous (2014) Orca Bay Seafoods, Inc. Renton: Orca Bay Foods, Inc.

10. Anonymous (2014) Premier Pacific Seafoods. Seattle: Premier Pacific Seafoods.

11. Anonymous (2014) Fishchoice.com - Helping business source and sell sustainable seafood. Fort Collins: FishChoice Inc.

12. Kingfisher (2014) Kingfisher Holdings Limited. Samutsakorn: Kingfisher Holdings Limited.

13. Anonymous (2014) A.K.Khan & Company Ltd. Chittagong: A.K.Khan & Company Ltd.

14. Nissui (2013) Nissui Environmental Report 2013. Tokyo: Nippon Suisan Kaisha, Ltd.

15. Nissui (2012) Nissui Company Profile. Tokyo: Nippon Suisan Kaisha, Ltd.

16. Nissui (2013) Supplemental Documents for 1st Quarter of Fiscal Year 2013. Tokyo: Nippon Suisan Kaisha.

17. Nissui (2013) Supplemental Documents for 2nd Quarter of Fiscal Year 2013. Tokyo: Nippon Suisan Kaisha.

18. Nissui (2013) Nissui Company Profile. Tokyo: Nippon Suisan Kaisha, Ltd.

19. Nissui (2013) Yellowtail Aquaculture Business Using Artificial Seedlings. Tokyo: Nissui.

20. Anonymous (2013) Annual report of the Unisea fleet cooperative year 2012. Seattle: Natural Resource Consultants, Inc.

21. Anonymous (2014) Supplemental documents for Fiscal Year 2013. Tokyo: Nippon Suisan Kaisha, Ltd.

22. Nissui (2013) Financial Results for the Year Ended March 31, 2013. Tokyo: Nippon Suisan Kaisha, Ltd.

23. Anonymous (2012) In for the long term - Sustainability report 2011 report highlights. Hong Kong: Pacific Andes.

24. Nissui (2014) Nissui. Tokyo: Nissui.

25. Nissui (2014) Nissui Frontier. Tokyo: Nissui.

26. Kyowa (2014) Kyowacom. Kyowa Fishery co., ltd.

27. UniSea (2014) UniSea. Redmont: UniSea.

28. Anonymous (2014) Glacier Fish Company. Seattle: Glacier Fish Company, L.L.C.

29. EMDEPES (2014) EMDEPES - Empresa de Desarollo Pesquero de Chile S.A. Santiago: Empresa de Desarollo Pesquero de Chile S.A.

30. Anonymous (2013) The World's Seafood Expert - by Thai Union Group. Bangkok: Thai Union Group.

31. TUF (2013) TUF Annual Report 2012. Samutsakhon: Thai Union Frozen Products PCL.

32. TUF (2013) 2012 Results Presentation, March 2013. Bangkok: Thai Union Frozen Products

33. Anonymous (2014) 2013 Results Presentation, 24 February 2014. Bangkok: Thai Union Frozen Products.

34. Anonymous (2012) 2011 Investor Presentation, 22 February 2012. Bangkok: Thai Union Frozen Products.

35. Anonymous (2014) Annual Report 2013 - Thai Union Frozen Products PCL. Samutsakhon, Thailand: Thai Union Group.

36. TUF (2014) TUF - Thai Union Frozen Products PCL. Samutsakorn: Thai Union Frozen Products PCL.

37. Anonymous (2013) Sustainable Seafood the Marine Harvest Way. Oslo: Marine Harvest.

38. Anonymous (2013) Annual Report 2012 - Leading the Blue Revolution. Oslo: Marine Harvest.

39. Anonymous (2011) Salmon feed and the use of wild fish ingredients in feed for farmed salmon. Marine Harvest: You Tube - https://<http://www.youtube.com/watch?v=CKQU8ex8ric&list=PL04AE951B28146337>.

40. IntraFish (2013) The IntraFish 150 Report. London: IntraFish Media.

41. Kerstens D (2013) Investing in seafood 2013. London: IntraFish Media.

42. Anonymous (2014) Annual Report 2013 - Leading the Blue Revolution. Oslo: Marine Harvest.

43. Anonymous (2013) Salmon Farming Industry Handbook 2013. Bergen: Marine Harvest.

44. Anonymous (2013) Capital Markets Day - Marine Harvest - New York, 1st of May 2013. Bergen: Marine Harvest.

45. Harvest M (2014) Marine Harvest. Bergen: Marine Harvest ASA.

46. Skretting (2013) Delivering Sustainable Feed Solutions for Aquaculture. Stavanger: Skretting.

47. Nutreco (2014) Feeding the Future - Integrated Report 2013. Amersfoort, The Netherlands: Nutreco.

48. Skretting (2010) Skretting Sustainable Procurement 2010 Raw materials - Annex V: Sustainable Procurement Policy for Marine Products. Amersfoort: Nutreco.

49. Skretting (2014) Annual Sustainability Report 2013. Stavanger: Skretting Group.

50. Skretting (2014) Skretting - a Nutreco company. Stavanger: Skretting AS.

51. nutreco (2014) nutreco. Amersfoort: nutreco.

52. Pescanova (2013) Pescanova, S.A. - Anexo V Análisis económico financiero de las sociedades del Grupo - Informe de la Administración Concursal. Deloitte Advisory, S.L.

53. Pescanova (2010) Grupo Pescanova - Investors Presentation. ￼Pontevedra: Pescanova S.A.

54. Pescanova (2012) Grupo Pescanova H1 2012. Pontevedra: Pescanova S.A.

55. BDO (2012) ￼Pescanova, S.A. y Sociedades Dependientes (Grupo Pescanova) Cuentas annuales consolidades e informe de gestíon consolidado correspondientes al ejercicio 2012 junto con el informe de auditoría de cuentas anuales. BDO International Limited.

56. de Sousa-Faro MF, Tamargo JV (2012) Grupo Pescanova Q1 2012 Financial Results Presentation. Pontevedra: Pescanova S.A.

57. de Sousa-Faro MF, Tamargo JV (2012) Grupo Pescanova 2011 Financial Results Presentation. Pontevedra: Pescanova S.A.

58. Pescanova (2012) Pescanova 2011 Annual Accounts. Pontevedra: Pescanova S.A.

59. Tallaksen E (2013) Pescanova’s fishing tentacles: 100,000t from Africa to South America. undercurrentnews. London: undercurrentnews.

60. Pescanova (2014) Pescanova USA. Coral Gables: Pescanova USA.

61. Pescanova (2014) Grupo Pescanova. Pontevedra: Pescanova, S.A.

62. KOFA (2012) Statistical year book of overseas fisheries 원양산업 통계연보 한국원양산업협회. Korea: Korea Overseas Fisheries Association (KOFA).

63. Dongwon (2011) Non-consolidated Financial Statements - December 31, 2010 and 2009 (With Independent Auditors´Report Thereon). Seoul: Dongwon Industries Co., Ltd.

64. Baek W (2012) Dongwon Industries. Seoul: KDB Daewoo Securities Research.

65. Dongwon (2014) Dongwon industries. Seoul: Dongwon Industries Co., Ltd.

66. Dongwon (2014) Dongwon. Seoul: Dongwon.

67. Austevoll (2013) Annual Report 2012. Storebø: Austevoll Seafood ASA.

68. Austevoll (2014) Annual Report 2013. Storebø: Austevoll Seafood ASA.

69. Møgster A, Drivenes BK (2013) 2013 Austevoll Seafood ASA Q1 - Financial presentation. Storebø: Austevoll Seafood ASA.

70. Møgster A, Drivenes BK (2013) 2013 Austevoll Seafood ASA Q3 - Financial presentation. Storebø: Austevoll Seafood ASA.

71. Austevoll (2010) Austevoll Seafood History. Storebø: Austevoll Seafood ASA.

72. Møgster A, Drivenes BK (2013) 2013 Austevoll Seafood ASA Q2 - Financial presentation. Storebø: Austevoll Seafood ASA.

73. Anonymous (2013) Årsrapport 2012 - Annual Report 2012. Ålesund: Norway Pelagic.

74. Austevoll (2014) Austevoll Seafood ASA. Storebø: Austevoll Seafood ASA.

75. Anonymous (2014) FoodCorp S.A. - Austevoll Seafood Company. Santiago: FoodCorp Chile.

76. Anonymous (2014) Norway Pelagic. Ålesund: Norway Pelagic AS.

77. Anonymous (2014) Austral - Quality for the world. Lima: Austral Group S.A.A.

78. Welcon (2014) Welcon AS. Welcon AS.

79. Lerøy (2014). Bergen: Lerøy.

80. Anonymous (2014) Br. Birkeland AS. Storebø: Br. Birkeland AS.

81. Egersund (2014) Egersund Fish of Norway. Egersund: Egersund Fisk AS.

82. Anonymous (2012) In for the long term - Sustainability Report 2011. Hong Kong: Pacific Andes.

83. Anonymous (2012) From Ocean to Plate - Annual Report 2011. Hong Kong: Pacific Andes International Holdings Limited.

84. Anonymous (2011) Pacific Andes International Holding Limited - Corporate Presentation May 2011. Hong Kong International Holdings Limited: Pacific Andes.

85. Anonymous (2013) Structured for Future Growth. Hong Kong: Pacific Andes International Holdings Limited.

86. CFG (2009) China Fishery Group Limited - Corporate Presentation September 2009. Hong Kong: China Fishery Group Limited.

87. Anonymous (2013) Expanding Access in Fishery Resources - Annual Report 2013. Hong Kong: China Fishery Group Limited.

88. CFG (2013) Annual Report 2012 - Where Efficiency Meets Responsibility. Hong Kong: China Fishery Group Limited.

89. CFG (2014) China Fishery Group Limited - Fact Sheet. Hong Kong: China Fishery Group Limited.

90. Anonymous (2014) Advancing our Momentum - Annual Report 2013. Hong Kong: Paciifc Andes Resource Development Limited.

91. CFG (2014) China Fishery Group Limited. Hong Kong: China Fishery Group Limited.

92. Pacific_Andes (2014) Pacific Andes International Holdings Limited. Hong Kong: Pacific Andes.

93. Copeinca (2014) Copeinca. Lima: Copeinca.

94. Trident (2012) the source. Seattle: Trident Seafoods.

95. Trident (2013) From the Source to the Plate®. Seattle: Trident Seafoods.

96. Trident (2014) Trident seafoods. Seatle: Trident Seafoods Corporation.

97. Kyokuyo (2013) Corporate profile. Tokyo: Kyokuyo Co., Ltd.

98. Anonymous (2013) Business Report - 90th Business report. Tokyo: Kyokuyo Co., Ltd.

99. Kyokuyo (2011) The Kyokuyo Group Environmental Policy. Tokyo: Kyokuyo Co., LTD.

100. Kyokuyo (2014) Qingdao Kyokuyo International Co. Ltd. Qingdao: Qingdao Kyokuyo International Co. Ltd.

101. Kyokuyo (2014) Kyokuyo Co., Ltd. Tokyo: Kyokuyo Co., Ltd.

102. Kyokuyo (2014) Kyokuyo Europe B.V. Schiphol: Kyokuyo Europe B.V.

103. Kyokuyo (2014) Kyokuyo America Corp. Seattle: Kyokuyo America Corporation.

104. Kyokuyo (2014) Kyokuyo Suisan. Kyokuyo Suisan co. ltd.

105. Anonymous (2014) K&U Enterprise Co., Ltd. Samutsakorn: K&U Enterprise Company Limited.

106. Anonymous (2010) Sustainable Salmon Feed: Marine Ingredients. Bergen: EWOS.

107. EWOS (2012) Fish Forever. Bergen: EWOS.

108. Cermaq (2013) Cermaq Annual Report 2012. Oslo: Cermaq.

109. EWOS (2014) Report to Bondholders – January-December 2013. Bergen: EWOS.

110. EWOS (2013) Fish Oil and Marine Omega-3 in Salmon Feed. Spotlight. Bergen: EWOS.

111. Wathne E, Beaumbusch B (2014) EWOS Group - Presentation to Bond holders, January – December 2013. Bergen: EWOS.

112. EWOS (2014) Annual Report 2013. Oslo: EWOS/Albain Holdco Norway AS Group.

113. EWOS (2014) A World Leader in Fish Nutrition. Bergen: EWOS.

114. Cermaq (2013) Integrated Annual and Sustainability Report 2012. Oslo: Cermaq.

115. EWOS (2014) EWOS. Bergen: EWOS.

116. CPF (2013) Lead the Way to Sustainability – Sustainable Development Report 2012. Bangkok: Charoen Pokphand Foods Public Company Limited.

117. CPF (2013) Kitchen Of the The World - CFP Annual Report 2012. Bangkok: Charoen Pokphand Foods Public Co., Ltd.

118. CPF (2014) Kitchen Of the The World -Annual Report 2013. Bangkok: Charoen Pokphand Foods Public Co., Ltd.

119. Han Meng T (2012) Charoen Pokphand Foods. Singapore: DMG & Partners Research Pte. Ltd.

120. Keeratipipatpong W (2014) Shrimp industry on the rebound. Bangkok Post. Bangkok.

121. CPF (2014) Charoen Pokphand Group. Bangkok: Charoen Pokphand Group.
