# Supplementary material for: Transnational Corporations as ‘Keystone Actors’ in Marine Ecosystems
Source: PLoS One. 2015 May 27;10(5):e0127533. doi: 10.1371/journal.pone.0127533 (PMC4446349; doi:10.1371/journal.pone.0127533)
Supplement: S2 Table — (DOCX) [file pone.0127533.s002.docx]

**Supporting Information Table S2.** Additional sources of information

| **Type** | **References/sources** |
| --- | --- |
| Country and region | Alaska [1-4], Japan [5, 6], Korea [7, 8], the Southern Ocean [9, 10], the North East Atlantic [11] |
| Species and group of species | tuna [12-17], shrimp [18], toothfish [9, 10], whitefish [19-21] |
| Global fishing industry reports | FAO [22-24], OECD [25], Intrafish [18, 26-32], Undercurrentnews [33], ICIJ [34], Rabobank [35], Globefish [36], M&A international [37] and the World Bank [38] |
| Regional Fisheries Management Organizations (RFMOs) | CCSBT [39], IATTC [40], ICCAT [41], IOTC [42], WCPFC [43], CCAMLR [44], CCBSP [45, 46], GFCM [46], NEAFC [47], NASCO [48], NAFO [49], SEAFO [50], SPRFMO [51] |
| Media sources | Intrafish [26], Undercurrentnews [52], Minato-Tsukiji [53], FIS [54], atuna [55], the Fish Site [56], Seafood Source [57], Food Business Review [58], Shrimp News International [59], The Center for Public Integrity [60] and Bloomberg [61] |

**References**

1. Anonymous. Report of the Akutan Catcher Vessel Association 2011. Seattle: Akutan Catcher Vessel Association, 2012.

2. Anonymous. Pollock Conservation Cooperative and High Seas Catchers´ Cooperative - Final Joint Annual Report 2012. Fairbanks and Seattle: Pollock Conservation Cooperative and High Seas Catchers´ Cooperative, 2013.

3. Anonymous. 2013 Alaska Seafood Annual Report Juneau: Alaska Seafood, 2013.

4. Ettefagh S. 2012 Final Report Unalaska Fleet Cooperative. Wrangell: Sylver Fishing Company, 2013.

5. Anonymous. Japan´s Fishery at a Glance. Tokyo: Japan Fisheries Agency, 2012.

6. Anonymous. FY2012 Trends in Fisheries/FY2013 Fishery Policy – White Paper on Fisheries: Summary. Tokyo: Japan Fishery Agencytoky, 2014.

7. Park J. Subsidising plunder - Korean Government´s support to its distant water fishing industry. Seoul: Greenpeance East Asia Seoul, 2013.

8. KOFA. Statistical year book of overseas fisheries원양산업 통계연보 한국원양산업협회. Korea: Korea Overseas Fisheries Association (KOFA), 2012.

9. CCAMLR. CCAMLR Statistical Bulletin, Vol. 26 Hobart: CCAMLR - Commission for the Conservation of Antarctic Marine Living Resources; 2014 [cited 2014 June 24]. Available from: https://<http://www.ccamlr.org/en/document/data/ccamlr-statistical-bulletin-vol-26>.

10. COLTO. COLTO Mt Hawthorn: Coalition of Legal Toothfish Operators Inc; 2014 [cited 2014 June 24]. Available from: <http://www.colto.org/>.

11. ICES. ICES - International Council for the Exploration of the Seas Copenhagen: International Council for the Exploration of the Seas; 2014 [cited 2014 June 24]. Available from: <http://www.ices.dk>.

12. Campling L, Havice E, Ram-Bidesi V. Pacific Island Countries, the Global Tuna Industry and the International Trade Regime - A Guidebook. Solomon Islands: The Pacific Islands Forum Fisheries Agency, 2007.

13. Hamilton A, Lewis A, McCoy MA, Havice E, Campling L. Market and Industry Dynamics in the Global Tuna Supply Chain. Solomon Islands: the Pacific Islands Forum Fisheries Agency, 2011.

14. Greenpeace. Out of line - The failure of the global tuna longline fisheries. Amsterdam: Greenpeace, 2013.

15. Grenpeace. The Hidden Secrets of Canned Tuna. Korea: Greenpeace, 2012.

16. Miyake MP, Guillotreau P, Sun C-H, Ishimura G. Recent developments in the tuna industry - Stocks, fisheries, management, processing, trade and markets. Rome: Food and Agriculture Organization of the United Nations, 2010.

17. Ozaki E. The Present & the Future of International Tuna Longline Fishing Industry. Organization for the Promotion of Responsible Tuna Fisheries, 2010.

18. Seaman T. The World´s Top 30 Farmed Shrimp Suppliers. Bergen: IntraFish Media AS, 2012.

19. AkerSeafoods. Industry handbook: The white fish industry 2012. Ålesund: Aker Seafoods ASA, 2013.

20. Seafish. Responsible Sourcing Guide: Hake. Grimsby: Seafish, 2013.

21. Seafish. Responsible Sourcing Guide: Alaska pollock. Grimsby: Seafish, 2013.

22. FAO. Fishery and aquaculture atatistics. Global capture production 1950-2012 (FishstatJ). Rome: FAO Fisheries and Aquaculture Department 2014.

23. FAO. The state of world fisheries and aquaculture - Opportunities and challenges. Rome: Food and Agriculture Organization on the United Nations, 2014.

24. FAO. FAO yearbook. Fishery statistics: aquaculture production/Annuaire. Statistiques des peches: production de l'aquaculture/Anuario. Estadisticas de pesca:produccion de acuicultura. Rome: Food and Agriculture Organization of the United Nations, 2014.

25. OECD. Globalisation in fisheries and aquaculture - Opportunities and challenges. Paris: Organisation for Economic Co-operation and Development, 2010.

26. IntraFish. IntraFish Bergen: IntraFish media; 2014 [cited 2014 June 17]. Available from: <http://www.intrafish.com/>.

27. IntraFish. The IntraFish 150 Report. London: IntraFish Media, 2013.

28. Kerstens D. Investing in seafood 2013. London: IntraFish Media, 2013.

29. Berger M. The sustainable seafood handbook. Norway: Intrafish Media, 2013.

30. Nadkarni A. Top 45 seafood companies: Americas. Bergen: IntraFish Media AS, 2012.

31. Tallaksen E. The Top 50 european seafood companies. Bergen: IntraFish Media AS, 2012.

32. IntraFish. Top 40 Asian seafood report. Bergen: IntraFish Media AS, 2012.

33. Undercurrentnews. World´s 100 largest seafood companies. London: Undercurrentnews, 2013.

34. ICIJ. Looting the seas. Washington, DC: Center for Public Integrity), 2012.

35. Rabobank. Spotlight on Seafood - An Investor´s Guide to the Global Marine Protein Industry. Utrecht: Rabobank International, 2011.

36. Asche F. Globefish research programme - Exchange rates and the seafood trade. Rome: Food and Agriculture Organisation of the United Nations, 2014.

37. Anonymous. The seafood industry: A sea of buyers fishing for M&A opportunities across the antire value chain. M&A International Inc., 2013.

38. Anonymous. FISH to 2030 - Prospects for fisheries and aquaculture. Washington DC: The World Bank, 2013 Contract No.: 83177.

39. CCSBT. Report of the Nineteenth Annual Meeting of the Commission. Takamatsu City: Commission for the Conservation of Southern Bluefin Tuna, 2012.

40. IATTC. Inter-American Tropical Tuna Commission 83rd meeting. La Jolla: Inter-American Tropical Tuna Commission, 2012.

41. ICCAT. Report for biennial period, 2012-13 Part I (2012) - Vol. 1. Madrid: Internaitonal Commission for the Conservation of Atlantic Tunas, 2013.

42. IOTC. Report of the Sixteenth Session of the Indian Ocean Tuna Commission. Fremantle: Indian Ocean Tuna Commission, 2012.

43. WCPFC. Ninth Regular Session of the Commission. Manilla: Western and Central Pacific Fisheries Commission, 2012.

44. CCAMLR. Report of the thirty-first meeting of the commission. Hobart: Commission for the Conservation of Antarctic Marine Living Resources, 2012 Contract No.: CCAMLR-XXXI.

45. CCBSP. Report of the 17th annual conference of the parries to the Convention on the Conservation and Management of Pollock Resources in the Central Bering Sea. Convention on the Conservation and Management of Pollock Resources in the Central Bering Sea, 2012.

46. GFCM. Report of the thirty-sixth session. Rome: FAO General Fisheries Commission for the Mediterranean, 2012 Contract No.: 36.

47. NEAFC. Report of the 31st annual meeting of the North-East Atlantic Fisheries Commission. London: North-East Atlantic Fisheries Commission, 2012.

48. NASCO. Report of the twenty-ninth annual meeting of the council. Edinburgh: North Atlantic Salmon Conservation Organization, 2012.

49. NAFO. Meeting Proceedings of the General Council and Fisheries Commission for 2012/2013. Nova Scotia: Northwest Atlantic Fisheries Organization, 2013.

50. SEAFO. Report of the 9th annual meeting of the Commission, 2012. Swakopmund: South East Atlantic Fisheries Organisation, 2012.

51. SPRFMO. First Commission Meeting of the South Pacific Regional Fisheries Management Organisation (SPRFMO). Wellington: South Pacific Regional Fisheries Management Organisation 2013.

52. Undercurrentnews. undercurrentnews - seafood business news from beneath the surface London: Undercurrent News Limited; 2014 [cited 2014 June 17]. Available from: <http://www.undercurrentnews.com/>.

53. Minato-Tsukiji. Minato-Tsukiji: Minato-Yamaguchi.Co.,Ltd.; 2014 [cited 2014 June 17]. Available from: <http://www.minato-tsukiji.com/>.

54. FIS. FIS - Sweden: Fish Info & Services Co.Ltd; 2014 [cited 2014 June 17]. Available from: <http://fis.com/>.

55. atuna. atuna: Atuna.com; 2014 [cited 2014 June 17]. Available from: <http://www.atuna.com/>.

56. Anonymous. The Fish Site Sheffield: 5m Publishing; 2014 [cited 2014 June 17]. Available from: <http://www.thefishsite.com/>.

57. SeafoodSource. SeafoodSource.com - Your Global Seafood Solution: Diversified Business Communications; 2014 [cited 2014 June 17]. Available from: <http://www.seafoodsource.com/en/>.

58. FBR. FBR - Food Business Review: Progressive Digital Media Group Plc 2014 [cited 2014 June 17]. Available from: <http://www.food-business-review.com/>.

59. Anonymous. Shrimp News International - Free News and Background Information on World Shrimp Farming 2014. Available from: <http://www.shrimpnews.com>.

60. Anonymous. The Center for Public Integrity: The Center for Public Integrity; 2014 [cited 2014 June 17]. Available from: <http://www.publicintegrity.org/>.

61. Bloomberg. Bloomberg: Bloomberg L.P.; 2014 [cited 2014 June 17]. Available from: <http://www.bloomberg.com/>.
