# Supplementary material for: Transnational Corporations as ‘Keystone Actors’ in Marine Ecosystems
Source: PLoS One. 2015 May 27;10(5):e0127533. doi: 10.1371/journal.pone.0127533 (PMC4446349; doi:10.1371/journal.pone.0127533)
Supplement: S3 Table — (DOCX) [file pone.0127533.s003.docx]

**Table S3.** **Stock volumes handled by the studied companies, including the estimated total wild catch and the total catch for a number of key segments in global seafood production.** The number of companies within parenthesis is the total number of companies known to be involved in the respective segment, but the combined volume by companies was based on the smaller number, in order to reduce the risk of overlap by the investigated companies. *** Represents the total volume caught or produced by the investigated companies (including the marine wild capture stocks included in the table, as well as additional wild species captured). *(expert)* denotes expert estimate.

|  | | **Stock value (USD/ton)** | **Total global production (ton)** | **Combined volumes of companies (ton)** | **No. companies** | **Combined share (%)** |
| --- | --- | --- | --- | --- | --- | --- |
| *Marine capture* |  |  | 79 705 910 ^[1]^ | 9–13 million* | 13 | 11 – 16 % |
| *Wild capture* | Alaska pollock | 3 300 ^[2]^ | 3 271 426 ^[1]^ | 1 314 000 | 4 (out of 5) | 40.2 % |
|  | Skipjack and Yellowfin tuna | 2 400 ^[1]^ | 4 147 543 ^[1]^ | 860 000 | 4 | 20.7 % |
|  | Toothfish | 12 000 ^(expert)^ | 24 796 ^[1]^ | 5 202 | 3 | 21.0 % |
|  | Namibian hake | 1 160 ^[1]^ | 159 286 ^[1, 3]^ | 30 182 | 1 | 18.9 % |
|  | Peruvian anchovy | 210 ^(expert)^ | 4 692 855^[1]^ | 1 116 899 | 2 (out of 5) | 23.8 % |
|  | NE Atlantic pelagics | 380 ^[1]^ | 4 519 640 ^[1, 4]^ | 1 024 000 | 1 (out of 5) | 22.7 % |
| *Aquaculture* | Salmon | 4 730 ^[1]^ | 2 145 873 ^[2]^ | 745 723 | 5 | 34.8 % |
|  | Whiteleg shrimp | 4 280 ^[1]^ | 3 178 721 ^[1]^ | 198 314 | 4 | 6.2 % |
|  | Bluefin tuna | 30 000 ^(expert)^ | 28 210 ^[5]^ | 10 813 | 3 | 38.3 % |
| *Reduction and feeds* | Fishmeal | 1 800 ^[2]^ | 4 535 000 ^[6]^ | 471 520 | 3 (out of 4) | 10.4 % |
|  | Fish oil | 1 500 ^[2]^ | 903 000 ^[6]^ | 122 420 | 3 (out of 4) | 13.6 % |
|  | Feeds | 950 ^[2]^ | 20 000 000 ^[2]^ | 4 285 087 | 5 | 21.5 % |

**References**

1. FAO. Fishery and aquaculture atatistics. Global capture production 1950-2012 (FishstatJ). Rome: FAO Fisheries and Aquaculture Department 2014.

2. Kerstens D. Investing in seafood 2013. London: IntraFish Media, 2013.

3. FishSource. FishSource - Status and environmental performance of fisheries worldwide: Sustainable Fisheries Partnership (SFP); 2014 [cited 2014 June 17]. Available from: <http://www.fishsource.com/>.

4. ICES. North sea sandeel in division IIIa and subarea IV. Copenhagen: 2013 Contract No.: 6.4.22.

5. Minato-Tsukiji. Minato-Tsukiji: Minato-Yamaguchi.Co.,Ltd.; 2014 [cited 2014 June 17]. Available from: <http://www.minato-tsukiji.com/>.

6. IFFO. IFFO - The marine ingredients organisation London: IFFO - The Marine Ingredients Organisation; 2014 [cited 2014 June 21]. Available from: <http://www.iffo.net/>.
