# Supplementary material for: Transnational Corporations as ‘Keystone Actors’ in Marine Ecosystems
Source: PLoS One. 2015 May 27;10(5):e0127533. doi: 10.1371/journal.pone.0127533 (PMC4446349; doi:10.1371/journal.pone.0127533)
Supplement: S4 Table — (DOCX) [file pone.0127533.s004.docx]

Table S4. The history and vertical integration of the selected thirteen companies

| **Company** | **Headquarter** | **History** | **Vertical integration** |
| --- | --- | --- | --- |
| Maruha Nichiro | Tokyo, Japan | Maruha (1880) and Nichiro (1906) have both engaged in whaling, salmon canning and tuna fishing. Both established Alaskan companies in the 1980s. The two companies merged in 2007, becoming the world largest seafood company | Fishing, aquaculture, processing, distribution and marketing. Main species includes Pollock, tuna, salmon, crab, cod and many other species. Is also engaged in meat production, pet foods and fine chemicals |
| Nippon Suisan Kaisha  (Nissui) | Tokyo, Japan | Activities started in 1911, including trawling and later whaling, North Sea fishing, salmon and krill fishing. South American subsidiaries were established from the 1970s and US subsidiaries were acquired from the 1990s | Fishing, aquaculture, processing, distribution and marketing. Main species include Pollock, tuna, crab, shrimp, and many other species. Is also engaged in fine chemicals production. |
| Thai Union Frozen Products | Samutsakorn,  Thailand | Established in 1973. Invested in leading US tuna brands (e.g., Starkist) in the 1990s and 2000s. Expanded Asian shrimp farming activities in the 2000s. Acquired leading European tuna brand (MW Brand) in 2010 | Tuna fishing, processing and canning, shrimp aquaculture, shrimp feeds and pet food. Frozen and canned seafood (e.g., mackerel and salmon) |
| Marine Harvest | Bergen, Norway | Pan Fish ASA (founded 1992), Fjord Seafood ASA (1996) and Marine Harvest N.V. (1965) merged in 2006 to form Marine Harvest | Salmon aquaculture and processing (e.g. in Norway, Chile, Scotland, Canada, Ireland and France). Currently integrating both downstream (processing) and upstream (fish feeds) |
| Dongwon Group | Seoul,  South Korea | Founded in 1969 and engaged in canned tuna in the 1980s. Acquired tuna brand Starkist in 2008 and a Senegalese tuna cannery in 2011 | Fishing, canning, processing and retailing. Focusing on sashimi grade and canned tuna, as well as krill |
| Skretting | Stavanger,  Norway | Established in 1899, with fish feeds production starting in 1963 | Produces feeds for salmon, shrimp and tilapia. Skretting is part of Nutreco, a leading animal nutrition and fish feed company |
| Pescanova | Pontevedra, Spain | Founded in 1960, engaging in fishing activities in South America and Southern Africa and expanded with new companies in these regions in the 1970s. Engaged in aquaculture since the 1980s | Fishing, aquaculture, processing, distribution |
| Austevoll Seafood | Storebø, Norway | Established in 1981 and started pelagic fishing in Chile in 1991, and increasingly also in Norway. Acquired South American and Norwegian subsidiaries in the 2000s | Fishing for small pelagics, fishmeal, fish oil and feeds production, salmon farming, distribution and sales |
| Pacific Andes | Hong Kong | Established in 1986 and started by sourcing multiple Chinese species for export to the USA and Europe. Acquired Chinese fish processing facility in the 1990s. Expanded in to fishing activities in the 2000s | Fishing, fishmeal production, sourcing, processing, logistics and marketing. Main species include Peruvian anchovy and other small pelagics, Pollock and other groundfish |
| EWOS | Oslo, Norway | Activities started in 1935, including feeds production for salmon. Established international subsidiaries (Scotland, Chile, Canada) in the 1970s and 1980s and a Vietnamese joint venture in the 2010s. Split from Cermaq in 2013 | Fish feeds production |
| Kyokuyo | Tokyo, Japan | Established in 1937, active in salmon fishing, deep sea trawling, purse seining, frozen food production. Established joint ventures (Europe, Thailand) in 2000s | Fishing, aquaculture (e.g. Bluefin tuna), sourcing, processing and marketing. Tuna, salmon, whitefish, shrimp and a range of other species |
| Charoen Pokphand Foods  (CP Foods) | Bangkok, Thailand | The company is registered in 1978 and initially produced animal feed. Engaged in aquaculture production from the late 1990s and has invested in e.g., Europe and East Asia and expanded in shrimp aquaculture and feeds in the 2000s. Established its own restaurant business in 2010s | Shrimp feeds and shrimp aquaculture, food production and retail. CP Foods is a major agro-industrial food producer where aquaculture comprise less than 20% of revenues |
| Trident Seafood | Seattle, USA | Established in 1973 and engaged in king crab, salmon, herring and ground fish (fishing and processing) in Alaska | Fishing and processing of crab, salmon, Pollock, cod and herring. Owns and operates multiple vessels and processing plants along the Pacific North West coast of the USA |
